# Neuroatypical “Moving Mirrors”: exploring the impact of camera movements on individuals with Autism Spectrum Disorders without intellectual disabilities.

**DOI:** 10.1192/j.eurpsy.2024.357

**Published:** 2024-08-27

**Authors:** B. Demartini, V. Nistico’, R. Del Giudice, F. Serio, G. Boido, G. Ingrosso, F. Lombardi, C. Sanguineti, V. Casula, A. Baccara, E. Chiudinelli, F. Vairano, F. M. Panzeri, M. Giori, P. M. Inghilleri di Villadauro, R. Faggioli, O. Gambini, T. Subini

**Affiliations:** ^1^UO Psichiatria 51 e 52, ASST Santi Paolo e Carlo, Presidio San Paolo; ^2^ Aldo Ravelli Research Center for Neurotechnology and Experimental Brain Therapeutics; ^3^Health Science Department, University of Milan; ^4^Department of Psychology, University of Milan Bicocca; ^5^Dipartimento di Beni Culturali e Ambientali, University of Milan, Milan, Italy

## Abstract

**Introduction:**

Neurofilmology is a young and evolving research field, at the intersection between neuroscience and movie experiences, that explores how the brain processes and responds to visual storytelling. It involves examining the cognitive and emotional effects of movies on viewers, including social cognition and perspective-taking aspects. However, up to date, these studies have focused only on the neurotypical population, hence constituting a considerable gap in the literature with respect to individuals with neuroatypical functioning.

**Objectives:**

Aim of this study was to investigate the experience of film viewing and its correlates in individuals with a diagnosis of Autism Spectrum Disorders (ASD).

**Methods:**

30 neurotypical individuals and 30 individuals with ASD without intellectual disabilities were asked to observe 12 short video clips of 3 seconds length, showing an agent grasping an object from a table, and filmed with three different camera techniques: Still, Steadycam, Zoom; for each clip, they were asked to respond to six question on a Visual Analogue Scale (0-100) designed to investigate participants’ potential feeling of involvement with the observed scene, their comfort with the different filming conditions, and their estimation of the ecological plausibility of the different types of camera movements.

**Results:**

Participants felt more involved watching videos filmed with a Steadycam, with respect to the Zoom and Still condition. Within the neurotypical group participants felt more comfortable when the camera was in motion (both Steadycam and Zoom condition) compared to the Still condition; no differences were found between conditions in the ASD group, as if they felt equally comfortable in every condition administered, regardless the filming technique.

**Conclusions:**

First, our results reinforce prior findings regarding the influence of different camera techniques on neurotypical individuals. Second, they add to the existing literature suggesting that individuals with ASD may exhibit differences in their subjective experiences related to empathizing with characters and immersing themselves as actors when the camera replicates naturalistic movements, resulting in a diminished overall fulfillment in the movie-watching process.

**Disclosure of Interest:**

None Declared

